# One-step creation of CMS lines using a *BoCENH3*-based haploid induction system in *Brassica* crop

**DOI:** 10.1038/s41477-024-01643-w

**Published:** 2024-03-18

**Authors:** Fengqing Han, Xiaoli Zhang, Yuxiang Liu, Yumei Liu, Hong Zhao, Zhansheng Li

**Affiliations:** 1 State Key Laboratory of Vegetable Biobreeding, Institute of Vegetables and Flowers, Chinese Academy of Agricultural Sciences, Beijing, China; 2 State Key Laboratory of Vegetable Biobreeding, Tianjin Academy of Agricultural Sciences, Tianjin, China; 3 Key Laboratory for Vegetable Biology of Hunan Province, Engineering Research Center for Horticultural Crop Germplasm Creation and New Variety Breeding, Ministry of Education, Hunan Agricultural University, Changsha, China; 4 Beijing Vegetable Research Center (BVRC), Beijing Academy of Agricultural and Forestry Sciences, National Engineering Research Center for Vegetables, Beijing, China

**Keywords:** Molecular engineering in plants, Plant breeding

## Abstract

Heterosis utilization in a large proportion of crops depends on the use of cytoplasmic male sterility (CMS) tools, requiring the development of homozygous fertile lines and CMS lines^1^. Although doubled haploid (DH) technology has been developed for several crops to rapidly generate fertile lines^2,3^, CMS lines are generally created by multiple rounds of backcrossing, which is time consuming and expensive^4^. Here we describe a method for generating both homozygous fertile and CMS lines through in vivo paternal haploid induction (HI). We generated in-frame deletion and restored frameshift mutants of *BoCENH3* in *Brassica oleracea* using the CRISPR/Cas9 system. The mutants induced paternal haploids by outcrossing. We subsequently generated HI lines with CMS cytoplasm, which enabled the generation of homozygous CMS lines in one step. The *BoCENH3*-based HI system provides a new DH technology to accelerate breeding in *Brassica* and other crops.

## Abstract (web summary)

Han et al. develop *BoCENH3* mutants which trigger paternal haploid induction in *Brassica oleracea*. On the basis of this haploid inducer line, a workable system is proposed for transferring cytoplasmic male sterility to broccoli inbred lines.

## Main

Heterosis utilization has been extensively applied in plant breeding and contributes greatly to crop yield^5^. One of the most important processes for heterosis utilization is to develop homozygous (true-breeding) lines, which requires 6–8 generations of selfing^2,6^. In addition, heterosis utilization in many crops including most *Brassica* crops, as well as millet, carrot and some rice, maize, soybean and sorghum crops, exploits cytoplasmic male sterility (CMS) as a pollination control tool for hybrid seed production, requiring another 6–8 rounds of backcrossing to generate homozygous CMS lines^4^. These selfing and backcrossing processes are time consuming and costly for most crops^7^.

Doubled haploid (DH) technology has broad application in generating true-breeding lines within 1–2 generations^8^. In vitro haploid induction (HI) methods such as anther culture and microspore embryogenesis are expensive and limited by genotype recalcitrance^9^. In vivo HI technology has commonly been used in maize breeding for several decades^10^. In recent years, due to the cloning of genes including *MTL/NLD/ZmPLA1*, *DMP*, *ZmPOD65*, *CENH3* and *ECS1/2* that are responsible for HI^3,11–13^, in vivo HI has been extended to several plant species including *Arabidopsis*, rice, wheat, tomato, rapeseed, cabbage and *Medicago truncatula*^2,7–9,14–16^.

The *Arabidopsis CENTROMERIC HISTONE3* (*CENH3*) gene encodes a centromere-identifying protein histone H3 variant^17^. Modification of this gene has been shown to induce mainly paternal haploid progeny upon outcrossing to the wild type (WT)^12,18^. However, the establishment of a *CENH3*-based system is relatively difficult because it requires modifications such as the use of GFP–tailswap chimaeric proteins, the expression of non-native *CENH3*, in-frame deletions, single amino acid substitutions and heterozygous *cenh3* null mutation (*+/cenh3*), while homozygous *cenh3* null mutation is lethal^8,19–22^. At present, although *CENH3* is conserved in both monocotyledonous and dicotyledonous plants, *CENH3*-based paternal HI is available only for *Arabidopsis*, maize and wheat^8,21,22^.

The paternal haploid inducer line has the advantage of transferring cytoplasm between different genotypes, as the cytoplasm is maternally inherited during crossing. The maize *indeterminate gametophyte1* (*ig1*) mutation induces the formation of androgenetic haploids with an HI rate of 1–3% (refs. ^23,24^). This *ig1* haploid inducer is routinely used by breeders to transfer CMS cytoplasms, such as C, S, SD, Vg, ME and CA, to inbred lines^23^. The *CENH3*-based haploid inducer also enables the transfer of cytoplasm among different ecotypes in *Arabidopsis*^8,25^, but it has not been employed to produce CMS lines.

We tested whether modification of the *CENH3* homologue can be used for paternal HI and whether a *CENH3*-based paternal inducer line with a CMS cytoplasm enables the creation of CMS lines through the exchange of cytoplasmic and nuclear genomes in broccoli (*Brassica oleracea* var. *italica*), a globally important vegetable crop.

Using the *Arabidopsis* CENH3 as query, we searched the broccoli HDEM genome with the Basic Local Alignment Search Tool for protein (BLASTP) and found only one CENH3 homologue BolC8t52879H (BoCENH3). Phylogenetic analysis revealed that BoCENH3 is highly similar to *Arabidopsis* CENH3 with 66% sequence identity, and they are assigned to the same subclade (Supplementary Fig. 1 and Supplementary Table 1). We cloned *BoCENH3* from the broccoli inbred line CX33 and detected its expression in various broccoli tissues by reverse transcription quantitative PCR(RT‒qPCR), which indicated that *BoCENH3* is highly expressed in pistils and young buds, especially in pistils at 24–72 h after pollination (Supplementary Fig. 2), consistent with what has been found for authentic *CENH3* genes in *Arabidopsis*, barley and wheat^19,21^.

**Fig. 1:**
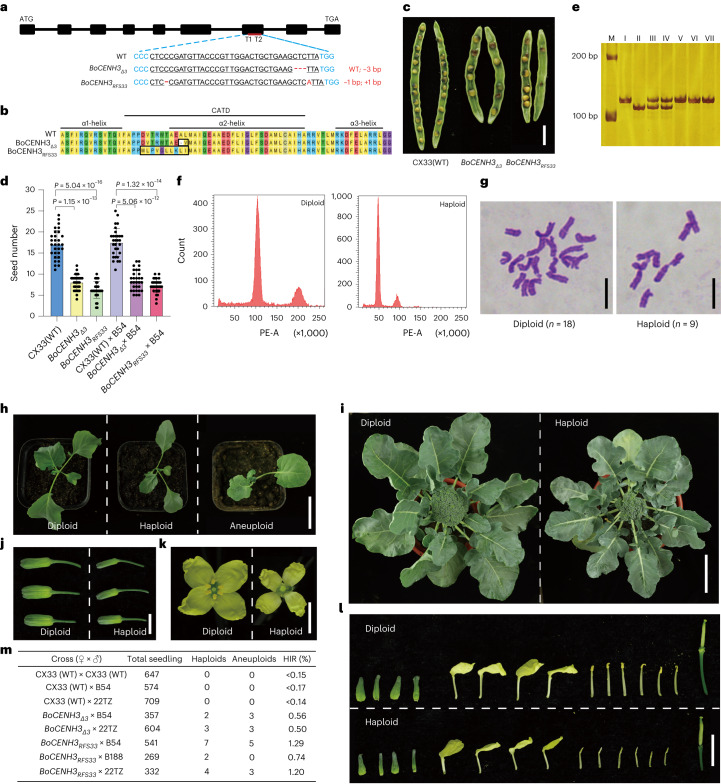
In-frame deletion and restored frameshift mutations of *BoCENH3* induce paternal haploids. **a**, Schematic diagram of *BoCENH3* gene structure and CRISPR/Cas9-based genome editing. Black blocks, gene coding region; red lines, sgRNA-targeted regions (T1, T2); underlining indicates the target sequence; protospacer adjacent motif sequences are highlighted in blue; insertions/deletions are highlighted in red. **b**, Amino acid alignment of BoCENH3, BoCENH3_Δ3_ and BoCENH3_RFS33_. Putative CATD and α-helix are indicated by black lines. Amino acid changes in *BoCENH3*_*Δ3*_ and *BoCENH3*_*RFS33*_ are outlined in black boxes. **c**, Representative siliques from selfed CX33 (WT) and *BoCENH3* mutants. **d**, Per silique seed setting performance of selfed WT, *BoCENH3*_*Δ3*_ and *BoCENH3*_*RFS33*_. Means ± s.d. (*n* = 30) (unpaired, two-tailed *t*-test). **e**, Haploid genotyping with DNA marker. M, DNA size marker; I–II, PCR bands of the B54 inbred line and the *BoCENH3*_*Δ3*_ mutant; III–IV, F_1_ hybrids from *BoCENH3*_*Δ3*_ × B54; V–VII, three haploids from B54. **f**, Flow cytometry analysis of diploid B54 and haploid B54 generated by *BoCENH3*_*Δ3*_ inducer. **g**, Chromosome numbers in diploid B54 (*n* = 18) and haploid B54 (*n* = 9) plants. **h**, Phenotypes of diploid, haploid and aneuploid B54 seedlings. **i**–**l**, Phenotypes of diploid and haploid B54 adult plants (**i**), buds (**j**), flowers (**k**) and dissected flower tissues (**l**). **m**, Haploid induction rates of *BoCENH3*_*Δ3*_ and *BoCENH3*_*RFS33*_. Scale bars, 1 cm (**c**), 10 µm (**g**), 5 cm (**h**), 10 cm (**i**), 0.5 cm (**j**,**k**) and 1 cm (**l**). Source data

We employed a CRISPR/Cas9 system to create hypomorphic alleles of *BoCENH3*. A CRISPR/Cas9 construct was generated with two specific guide RNA sequences (sgRNA1 and sgRNA2) targeting the 7th exon of *BoCENH3*, located within the sequence encoding the putative α2-helix region^26^ (Fig. 1a,b). The construct was subsequently introduced into the inbred line CX33, after which 21 independent transgenic lines were obtained. Among these transgenic lines, 11 lines harboured mutations in either of the targeted regions (3 for both sgRNA1 and sgRNA2, and 8 for only sgRNA2), corresponding to editing efficiencies of 14.29% for sgRNA1 and 52.38% for sgRNA2 (Supplementary Table 2). Eight lines were heterozygous or chimaeric mutants with deletions/insertions that led to frameshift and premature termination. Although not homozygous mutants, they displayed wrinkled leaves, a typical feature of defects in cell division (Supplementary Fig. 3). No homozygous mutants were produced from the T0 genome editing lines or selfing of T0 lines, consistent with the previous reports that *CENH3* is essential for plant development and reproduction^12,26^. Two representative heterozygous (*BoCENH3*/*bocenh3*) mutants were crossed by pollen donors, the broccoli lines B54 and 22TZ, and 2,512 progenies from four crosses were assessed (Supplementary Table 2). Although the heterozygous *CENH3* mutant (*+/cenh3*) with a null allele triggered haploid production in maize^22^, the broccoli heterozygous (*BoCENH3*/*bocenh3*) mutants did not induce haploids at the scale tested here (zero haploid in a screen of ~600 plants, for an average HI frequency of <0.16). The HI rate in lines homozygous for WT *CENH3* is probably considerably lower than this upper limit, as in a test for spontaneous paternal haploid in the related species *A. thaliana*, paternal haploids were not observed in a population of over 800,000 F_1_ progenies (<1 × 10^−6^)^27^. The remaining two lines, #3 and #8, harboured in-frame deletions in targeted region 2 and a restored frameshift (RFS) between the two targeted regions, respectively (Fig. 1a,b and Supplementary Table 2). From the T1 generation, we generated non-transgenic homozygous mutants *BoCENH3*_*Δ3*_ (*BoCENH3*_*Δ3*_/*BoCENH3*_*Δ3*_) and *BoCENH3*_*RFS33*_ (*BoCENH3*_*RFS33*_/*BoCENH3*_*RFS33*_), which exhibited a normal appearance compared with that of WT plants. When selfing or crossing as a female parent, the *BoCENH3*_*Δ3*_ and *BoCENH3*_*RFS33*_ mutants showed significantly reduced seed setting rates (*P* < 0.01; unpaired, two-tailed *t*-test) (Fig. 1c,d).

To test whether the *BoCENH3*_*Δ3*_ and *BoCENH3*_*RFS33*_ mutants could induce haploids, these mutants were crossed by 3 pollen donors: B54, B188 and 22TZ. These pollen donors were broccoli inbred lines with distinct genetic backgrounds and phenotypes (Supplementary Fig. 4). We developed 9 molecular markers (one for each chromosome) showing insertion–deletion (indel) polymorphisms between the mutants and the male parents to screen all the progenies. Potential haploids that exhibited genotypes identical to those of the male parents were identified (Fig. 1e and Supplementary Fig. 5). Flow cytometry analyses and chromosome spreads confirmed that all the plants were true haploids (Fig. 1f,g). These haploids were morphologically similar to the corresponding male parents but had smaller plant sizes, thinner leaves and male sterility (Fig. 1h–l). The HI rate was 0.52% on average for *BoCENH3*_*Δ3*_ and 1.14% for *BoCENH3*_*RFS33*_, substantially higher than that observed in selfed WT plants (0 in 647 progenies, HI rate < 0.15%), WT × B54 crosses (0 in 574 progenies, HI rate < 0.17%) and WT × 22TZ crosses (0 in 709 progenies, HI rate < 0.14%) (Fig. 1m). Nevertheless, this HI rate is relatively lower than the previously reported 1–44% in *Arabidopsis* (varied in different *CENH3* mutants)^26^, ~7% in wheat (in-frame modification of the N-terminal domain)^21^ and 5% in maize (heterozygous mutant with a *cenh3* null allele)^22^. The generation of additional types of *BoCENH3* mutants may be helpful in increasing the HI rate in broccoli. In addition, we identified aneuploids from the outcrossing progenies (Fig. 1h,m and Supplementary Fig. 6). Given that some *CENH3* mutants could induce both paternal maternal and maternal haploids^26^, we tested whether the *BoCENH3* mutants could trigger maternal haploids in reciprocal crosses. Although the seed setting rate was also slightly reduced when these two mutants were crossed as males, no haploids were identified at the scale (2,699 individuals) tested here (Supplementary Table 4).

**Fig. 2:**
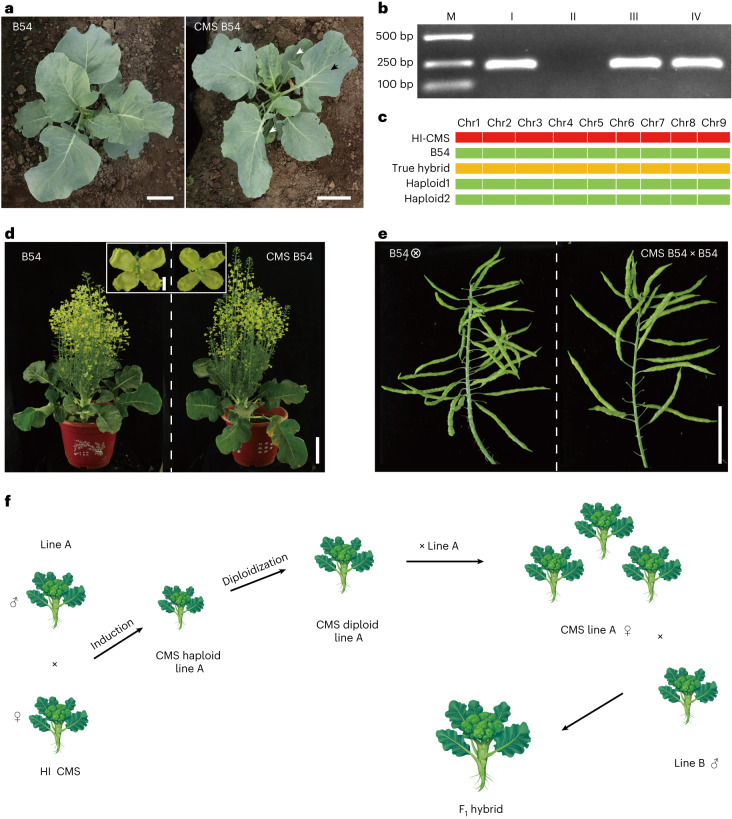
One-step creation of CMS lines using the *BoCENH3*_*RFS33*_ inducer line with an Ogura CMS cytoplasm. **a**, Phenotypes of WT B54 and doubled haploid CMS B54 seedlings. White arrows indicate haploid leaves before diploidization with colchicine and black arrows indicate diploid leaves after treatment with colchicine. **b**, PCR confirmation of the cytoplasm type using an *orf138* specific marker. M, DNA size marker; I, Ogura CMS line; II, inbred line B54; III–IV, B54 haploids with Ogura CMS cytoplasm. Three independent experiments were performed with similar results. **c**, Genetic background of the HI-CMS line, B54, F_1_ hybrid and haploid revealed by DNA markers on 9 chromosomes. **d**,**e**, Phenotype (**d**) and seed setting performance (**e**) of the CMS-B54 line. **f**, A proposed model of an effective CMS-based heterosis utilization system. Scale bars, 5 cm (**a**), 10 cm (whole plant in **d**), 5 cm (single flower in **d**) and 5 cm (**e**). Source data

Almost all commercial *B. oleracea* hybrids are produced on the basis of the Ogura CMS system, which requires the creation of CMS lines^28^. Currently, CMS lines in *Brassica* crops are exclusively developed through the traditional backcrossing procedure^28,29^. Although swapping of CMS via a paternal HI line (*ig1*) is already used in maize^23^, this strategy has not been extended to other species. We tested whether the CMS cytoplasm can be exchanged to inbred lines via *BoCENH3*-based HI. To create HI lines in the Ogura CMS cytoplasm, the CMS line CMS219 was crossed with *BoCENH3*_*RFS33*_ pollen and then backcrossed to *BoCENH3*_*RFS33*_. From the BC_1_ generation, we identified homozygous *BoCENH3*_*RFS33*_ individuals with Ogura CMS cytoplasm (harbouring the *orf138* gene)^28^. The HI-CMS line was crossed with a B54 pollen donor. We identified 5 haploids from 492 progenies (1.02%) using molecular markers, flow cytometry and plant phenotype analyses (Fig. 2a and Supplementary Fig. 7). Using the *orf138* specific gene marker and nuclear genomic markers, we confirmed that these haploids had a B54 genetic background and Ogura CMS cytoplasm (Fig. 2b,c). These CMS B54 plants were treated with 200 mg l^−1^ colchicine solution for chromosome doubling. We successfully obtained two diploid CMS-B54 lines, which were morphologically similar to its maintainer B54 and showed typical Ogura male sterile traits (Fig. 2a,d and Supplementary Fig. 8). When crossed to its maintainer line, CMS-B54 exhibited normal seed setting performance (Fig. 2e and Supplementary Fig. 9). To further confirm the genomic background of the haploids and diploids, the parental lines, two diploid CMS B54 plants and six B54 haploids were subjected to whole-genome resequencing. More than 318,000 single-nucleotide polymorphisms (SNPs) were identified from the samples. Most SNPs (>99.5%) from B54 CMS lines and B54 haploids showed genotypes identical to the original B54 line and no credible maternally derived SNPs were found (Supplementary Table 5), indicating that *BoCENH3*_*RFS33*_ could induce clean paternal haploids.

In summary, we successfully established a *CENH3*-based paternal HI system in broccoli, providing a novel and cost-effective DH technology without genotype recalcitrance. Importantly, by creating paternal haploids with maternal cytoplasm, homozygous CMS lines can be created in one step, which is a breakthrough for crops using CMS systems for hybrid seed production (a proposed system is shown in Fig. 2f). The success of this HI system could accelerate the improvement of *B. oleracea* cultivars and pave the way for extending *CENH3*-based HI to other crops.

## Methods

### Plant materials

The broccoli inbred line CX33 was employed for the purpose of genetic transformation and *BoCENH3* knockout. The inbred lines B54, B188 and the 22TZ were used as test lines for HI of *BoCENH3* inducers. The broccoli CMS line CMS219 was used as a donor of Ogura cytoplasm. All plants and the derived transgenic plants were grown under local conditions in Beijing, China. All selfing and cross-pollinations were performed in a greenhouse.

### RNA isolation and RT‒qPCR analyses

Fresh broccoli tissues, including leaves, stems, roots, floral buds, petals, sepals, mature pollens, pistils and siliques were sampled and submerged in liquid nitrogen. An RNA extraction kit (Tiangen Biotech, 4992239) was used for total RNA isolation from these broccoli tissues. A complementary (c)DNA Synthesis kit (Tiangen, 4992910) was used for synthesizing cDNA following standard protocol. For qPCR assays, we prepared 25 μl PCR mixture using SYBR Green master mix (Takara, RR82WR) and performed PCR experiments on an RT–qPCR detection platform (CFX96 Touch, Bio-Rad). As an internal control, RT‒qPCR for the *Actin* gene of *B. oleracea* was also performed alongside the experimental group. Each assay was biologically repeated at least three times. The data analysis was performed using the 2^−ΔΔCT^ method^30^.

### Phylogenetic analyses

The protein sequence of BoCENH3 was downloaded from the broccoli draft genome HDEM (https://www.genoscope.cns.fr/projet_BKL/cgi-bin/gbrowse/boleracea/)^31^. To search for proteins homologous to this sequence, we utilized the BLASTP tool in Ensembl Plants (http://plants.ensembl.org/index.html) with BoCENH3 protein sequence as a query. A total of 12 BoCENH3 homologue proteins from 10 species were used for phylogenetic analyses. The protein sequences were subjected to alignment through ClustalW in MEGA software (v.7) and then a neighbour-joining phylogenetic tree was constructed using MEGA (Poisson model, 1,000 bootstrap replicates). Sequences of the homologous proteins used in this study are shown in Supplementary Table 1.

### Genetic transformation and CRISPR/Cas9-mediated gene editing in broccoli

The sequence of *BoCENH3* from broccoli CX33 was used for sgRNA design. We searched for the conserved motifs of α-helix and centromere-targeting domain (CATD) in *BoCENH3* by comparing it to the known *Arabidopsis CENH3* (ref. ^26^). Two sgRNA sequences targeting the putative α2-helix region were chosen and inserted into a modified vector downstream of the *Arabidopsis* U6 promoter. We further cloned and inserted this cassette to a vector with a CaMV 35S promoter-derived *Bar* selection marker and a CaMV 35S promoter-derived *Cas9* gene as previously described^32^. The protocol for *Agrobacterium*-mediated broccoli transformation using the material CX33 has been described previously^32,33^. Positive transgenic plants were screened by Basta resistance and *Bar* gene specific marker.

The genomic fragment encompassing the sgRNA-targeted regions was amplified from positive transgenic plants and subjected to Sanger sequencing. To determine the mutations in T0 plants, PCR amplicons from these lines with expected mutations in *BoCENH3* were inserted into a cloning vector, introduced into *Escherichia coli* strain *EH5*α and then sequenced (at least 20 independent clones for each line)^32^. Homozygous in-frame deletion and restored frameshift mutants of *BoCENH3* in the T1 or BC_1_ generations were confirmed by PCR and Sanger sequencing.

### Whole-genome resequencing and genotyping

Genomic DNA was extracted from leaves of B54, *BoCENH3*_*RFS33*_ haploid inducer, two B54 CMS lines and six B54 haploids. DNA libraries were constructed and then subjected to whole-genome sequencing using the DNBSEQ-T7 platform. Low-quality reads were filtered using Trimmomatic software and the retained clean reads were processed with the BWA software by aligning the reads to the broccoli HDEM draft genome. Uniquely mapped reads were employed in the process of whole-genome SNP calling via GATK4 following the pipeline for GATK best practices. High-quality SNPs were selected and filtered following the reported parameters^34^. Only sites where genotyping was available for all accessions were retained.

### Haploid screening

The progenies of the *BoCENH3* mutants × broccoli lines were analysed by genetic background, flow cytometry, chromosome number and plant phenotype analyses. For genetic background analyses, markers were developed on the basis of indel variations between the *BoCENH3* HI inducer and broccoli test lines, and used to genotype all the progenies. For potential haploids identified by molecular markers, flow cytometry and chromosome number analyses were performed following previously described protocols^35^.

To determine the cytoplasmic background of the haploids or DH individuals, PCR was performed using an *orf138* (the causal gene of Ogura CMS) specific marker. Information on all primers used in the study can be found in Supplementary Table 6.

### Chromosome doubling

To generate DH lines, the identified CMS B54 haploid seedlings at the stage of 3–4 true leaves were treated with 200 mg l^−1^ colchicine solution by root dip treatment for 20 h. The ploidy levels of plants were detected after treatment.

### Phenotyping and statistical analyses

Images of plant tissues were acquired using a digital camera (EOS M6, Canon). Subsequent processing of these images was conducted using Adobe Photoshop CS6 (v.13.0) and Adobe Illustrator 2022 (v.26.2). Statistical analyses were conducted using GraphPad Prism (v.9) and Microsoft Excel (2019).

### Reporting summary

Further information on research design is available in the Nature Portfolio Reporting Summary linked to this article.

### Supplementary information

Supplementary Information Supplementary Figs. 1–9. Unprocessed gels for Supplementary Fig. 5.

Reporting Summary

Supplementary Tables Supplementary Tables 1–6.

Supplementary Data Statistical source data for Supplementary Figs. 2 and 9.

### Source data

Source Data Fig. 1 Statistical source data for Fig. 1d.

Source Data Fig. 1 Unprocessed gels for Fig. 1e.

Source Data Fig. 2 Unprocessed gels for Fig. 2b.

## Data Availability

The whole-genome resequencing data used in this study are accessible in NCBI through the accession code PRJNA1050660. The broccoli HDEM reference genome is publicly available (https://www.genoscope.cns.fr/projet_BKL/cgi-bin/gbrowse/boleracea/). CENH3 homologue proteins can be obtained from the database Ensembl Plants (http://plants.ensembl.org/index.html). Source data are provided with this paper.
