# Supplementary material for: One-step creation of CMS lines using a BoCENH3-based haploid induction system in Brassica crop
Source: Nat Plants. 2024 Mar 18;10(4):581–6. doi: 10.1038/s41477-024-01643-w (PMC11035129; doi:10.1038/s41477-024-01643-w)
Supplement: Supplementary file 1 — Supplementary Figs. 1–9. Unprocessed gels for Supplementary Fig. 5. [file 41477_2024_1643_MOESM1_ESM.pdf]

nature plants

Letter

https://doi.org/10.1038/s41477-024-01643-w

One-step creation of CMS lines using a *BoCENH3*-based haploid induction system in *Brassica* crop

In the format provided by the authors and unedited

Contents

Supplementary Figures 1–9.

Unprocessed gels for Supplementary Figure 5.

SUPPORTING INFORMATION

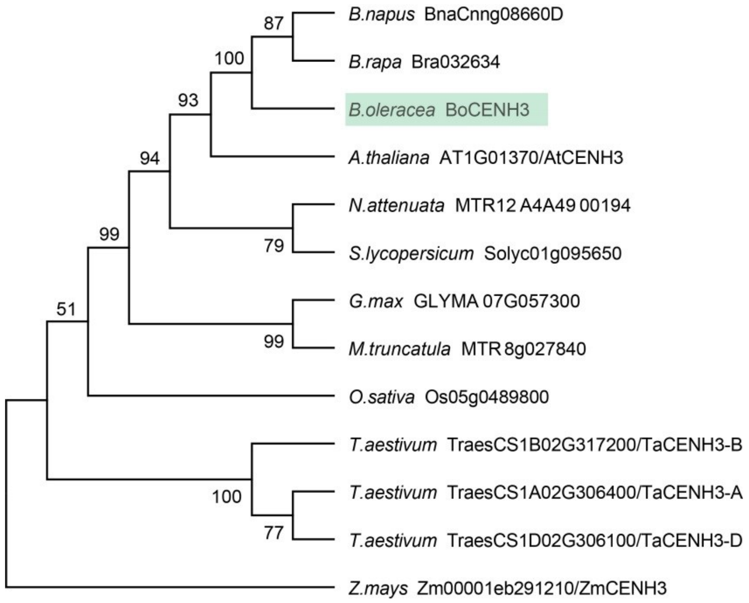

Supplementary Figure 1. Phylogenetic analysis of CENH3 homologs in *Brassica oleracea*, *Brassica napus*, *Nicotiana attenuate*, *Solanum lycopersicum*, *Glycine max*, *Oryza sativa*, *Medicago truncatula*, Zea *mays* and *Triticum aestivum*. BoCENH3 are indicated in green block. A neighbour-joining phylogenetic tree (1000 bootstrap replications) was constructed using MEGA (version 7) software.

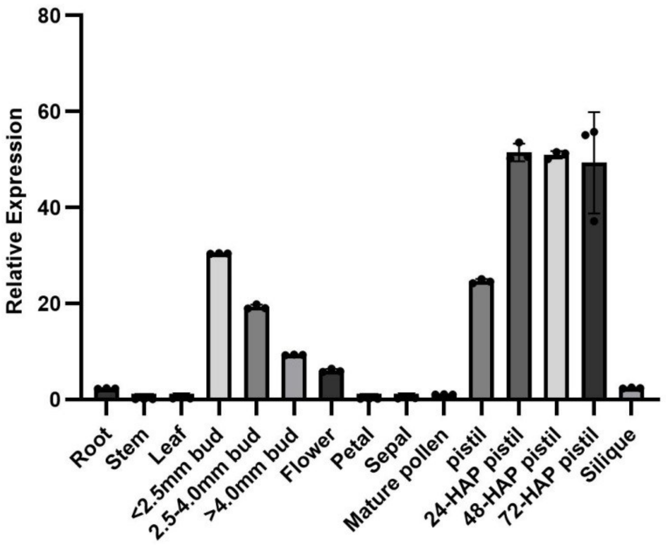

Supplementary Figure 2. Expression patterns of *BoCENH3* in various broccoli tissues. Three independent experiments were performed with similar results. The error bars are represented as the means ± SD (n = 3). HAP, hours after pollination.

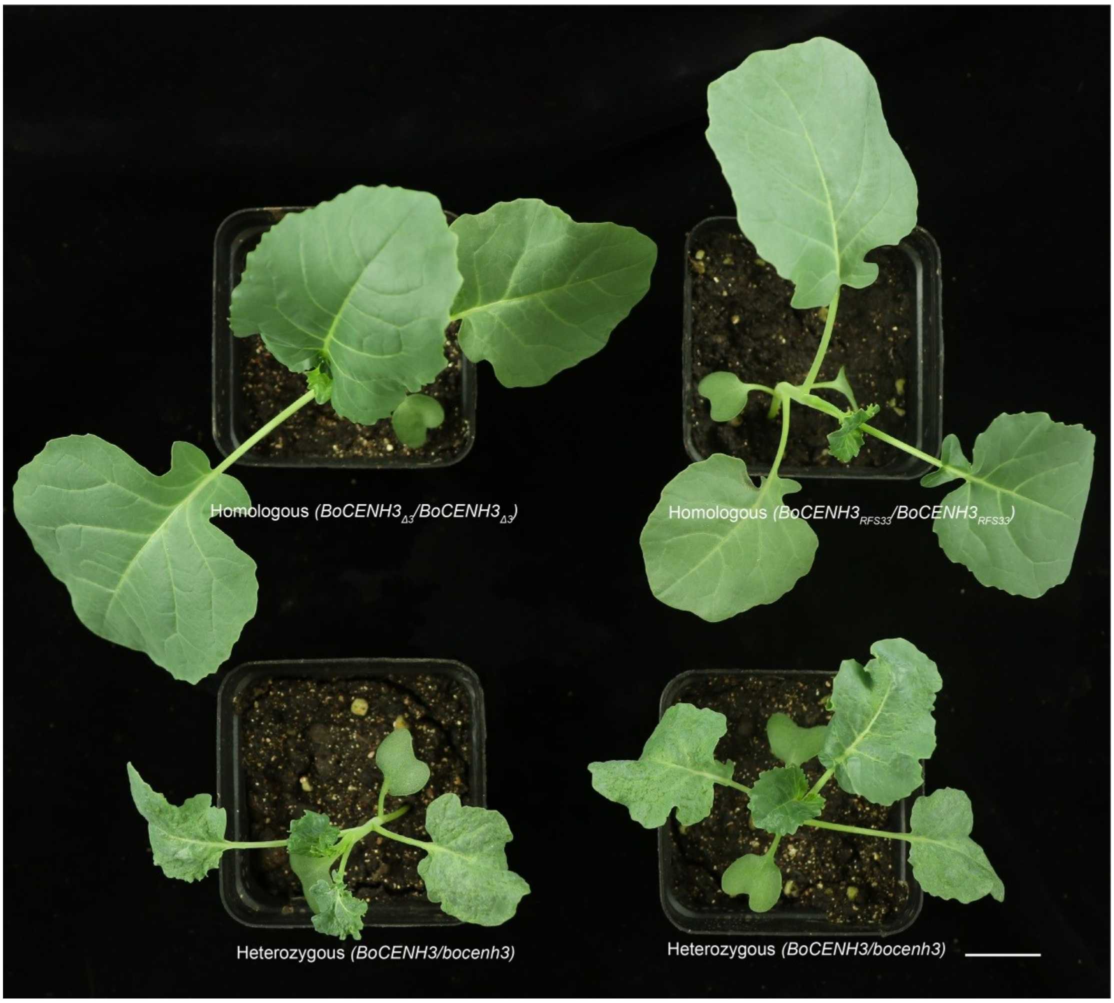

Supplementary Figure 3. Phenotype of in frame deletion mutant *BoCENH3*$_{\Delta 3}$ (*BoCENH3*$_{\Delta 3}$/*BoCENH3*$_{\Delta 3}$), restored frameshift mutant *BoCENH3*$_{RFS33}$ (*BoCENH3*$_{RFS33}$/*BoCENH3*$_{RFS33}$), and heterozygous mutants (*BoCENH3*/*bocenh3*). Scale bar = 2 cm.

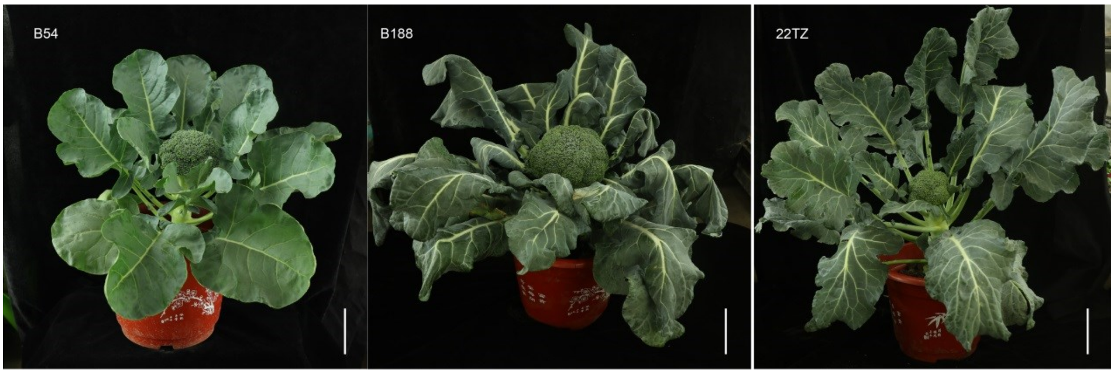

Supplementary Figure 4. Phenotypes of pollen donors B54, B188 and 22TZ. Scale bar = 10 cm.

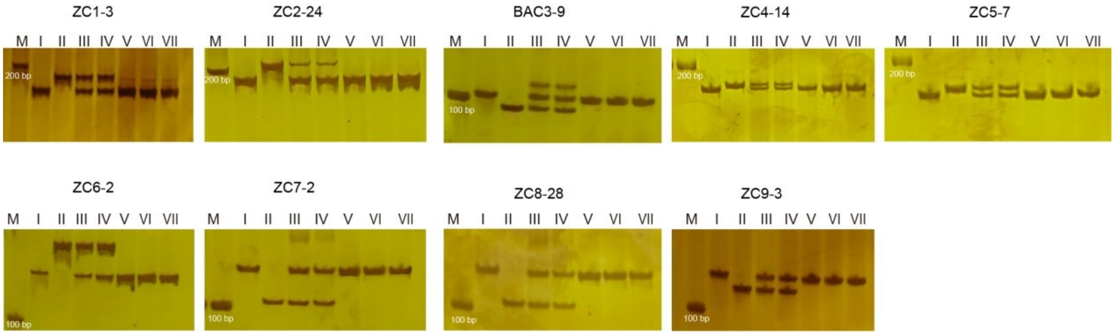

Supplementary Figure 5. Haploid genotyping with DNA markers on 9 chromosomes. ZC1-3, ZC2-24, BAC3-9, ZC4-14, ZC5-7, ZC6-2, ZC7-2, ZC8-28 and ZC9-3 are markers on chromosomes C1~C9. M, DNA size marker; I-II, PCR bands of the B54 inbred line and *BoCENH3*$_{\Delta 3}$ mutant; III-IV, F_1 hybrids from *BoCENH3*$_{\Delta 3}$ × B54; V-VII, three haploids from B54. Genotyping experiments were repeated for at least three times with similar results.

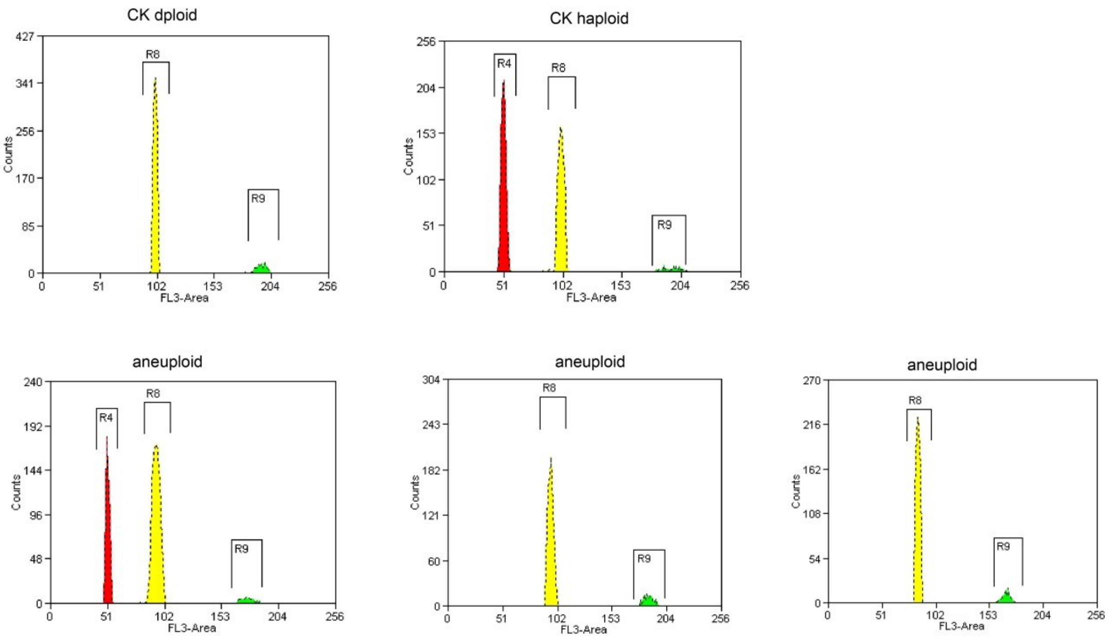

Supplementary Figure 6. Flow cytometry analysis of diploid B54, haploid B54 and three aneuploids generated by the *$BoCENH3_{\Delta 3}$* inducer.

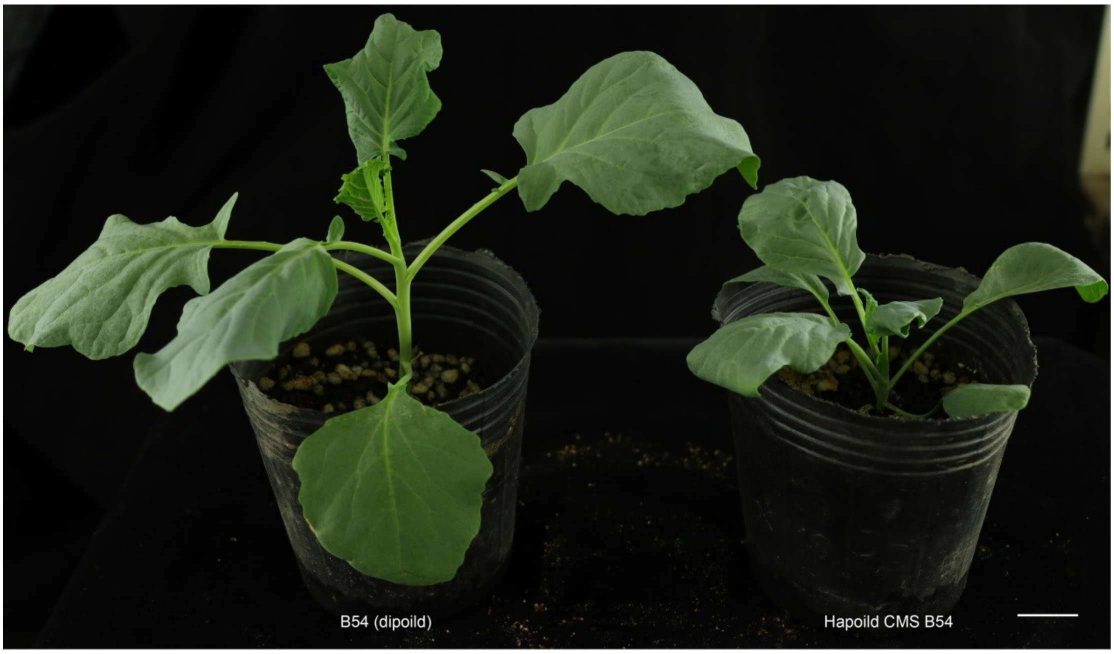

Supplementary Figure 7. Phenotypes of wild type B54, and B54 haploid with Ogura CMS cytoplasm induced by the HI-CMS line. Scale bar = 2 cm.

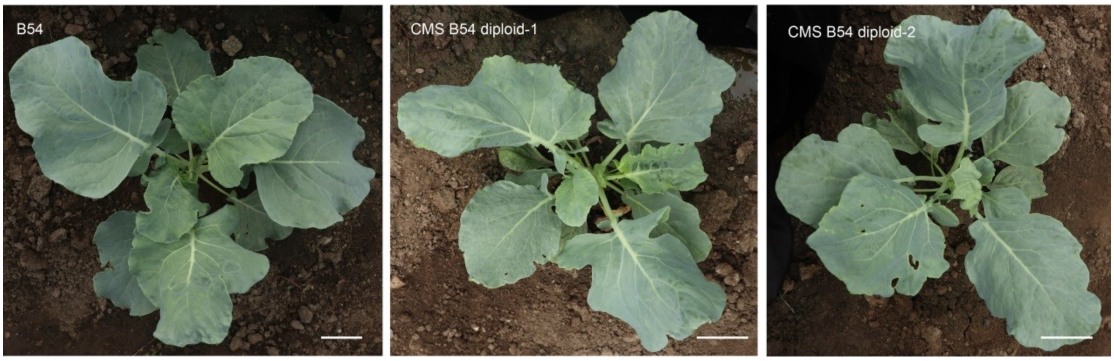

Supplementary Figure 8. Phenotypes of wild type B54, and two doubled haploid CMS B54 seedlings. Scale bars = 5 cm.

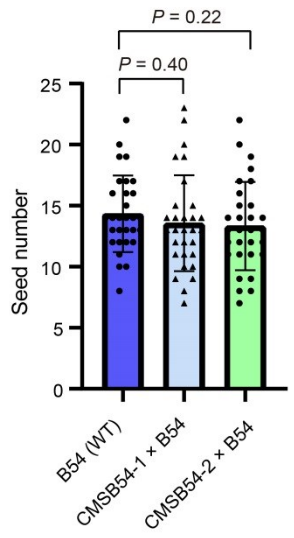

Supplementary Figure 9. Per silique seed setting performance of selfed wild type B54 and two B54 CMS lines generated by *BoCENH3*-mediated HI. Bars represent the means ± SD (n = 30); Two-tailed unpaired *t*-test was used for statistical analysis.

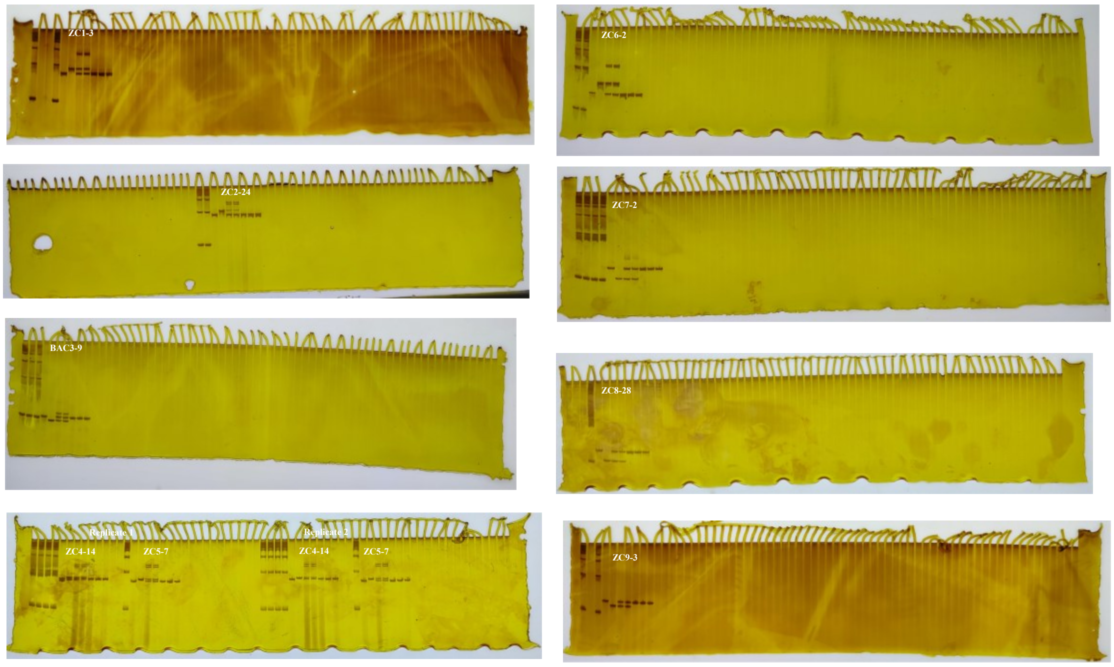

Unprocessed gels for Supplementary Figure 5.
