# Supplementary material for: One-step creation of CMS lines using a BoCENH3-based haploid induction system in Brassica crop
Source: Nat Plants. 2024 Mar 18;10(4):581–6. doi: 10.1038/s41477-024-01643-w (PMC11035129; doi:10.1038/s41477-024-01643-w)
Supplement: Supplementary file 2 — Reporting Summary [file 41477_2024_1643_MOESM2_ESM.pdf]

nature portfolio

Corresponding author(s): Zhansheng Li

Last updated by author(s): Jan 26, 2024

Reporting Summary

Nature Portfolio wishes to improve the reproducibility of the work that we publish. This form provides structure for consistency and transparency in reporting. For further information on Nature Portfolio policies, see our Editorial Policies and the Editorial Policy Checklist.

Statistics

For all statistical analyses, confirm that the following items are present in the figure legend, table legend, main text, or Methods section.

n/a	Confirmed	
☐	☒	The exact sample size (*n*) for each experimental group/condition, given as a discrete number and unit of measurement
☐	☒	A statement on whether measurements were taken from distinct samples or whether the same sample was measured repeatedly
☐	☒	The statistical test(s) used AND whether they are one- or two-sided<br>*Only common tests should be described solely by name; describe more complex techniques in the Methods section.*
☒	☐	A description of all covariates tested
☒	☐	A description of any assumptions or corrections, such as tests of normality and adjustment for multiple comparisons
☐	☒	A full description of the statistical parameters including central tendency (e.g. means) or other basic estimates (e.g. regression coefficient) AND variation (e.g. standard deviation) or associated estimates of uncertainty (e.g. confidence intervals)
☐	☒	For null hypothesis testing, the test statistic (e.g. *F*, *t*, *r*) with confidence intervals, effect sizes, degrees of freedom and *P* value noted<br>*Give P values as exact values whenever suitable.*
☒	☐	For Bayesian analysis, information on the choice of priors and Markov chain Monte Carlo settings
☒	☐	For hierarchical and complex designs, identification of the appropriate level for tests and full reporting of outcomes
☒	☐	Estimates of effect sizes (e.g. Cohen's *d*, Pearson's *r*), indicating how they were calculated

Our web collection on statistics for biologists contains articles on many of the points above.

Software and code

Policy information about availability of computer code

Data collection	In this study,no custom-made software or codes/mathematical algorithms were generated by the authors. Whole-genome resequencing data were generated using the high-throughput sequencing platform DNBSEQ-T7. Plant tissues were captured with a Canon EOS M6 digital camera (Canon, Tokyo, Japan) .
Data analysis	Statistical analyses were conducted using GraphPad Prism (Version 9) and and Microsoft Excel (Version 2019). Protein sequences were aligned by ClustalW in MEGA software (version 7). Phylogenetic tree was constructed with MEGA software (version 7). Whole-genome resequencing data were processed with GATK (Version 4.1.4.0), Trimmomati (Version 0.39) and BWA (Version 0.7.17). Images were processed with Adobe Illustrator 2022 (version 26.2) and Adobe Photoshop CS6 (version 13.0).

For manuscripts utilizing custom algorithms or software that are central to the research but not yet described in published literature, software must be made available to editors and reviewers. We strongly encourage code deposition in a community repository (e.g. GitHub). See the Nature Portfolio guidelines for submitting code & software for further information.

Data

Policy information about availability of data

All manuscripts must include a data availability statement. This statement should provide the following information, where applicable:

- Accession codes, unique identifiers, or web links for publicly available datasets
- A description of any restrictions on data availability
- For clinical datasets or third party data, please ensure that the statement adheres to our policy

The genomic resequencing data generated in this study have been deposited at NCBI under accession PRJNA1050660. Broccoli HDEM reference genome is public available (https://www.genoscope.cns.fr/projet_BKL/cgi-bin/gbrowse/boleracea/). CENH3 homolog proteins can be obtained from the database Ensembl Plants (http://plants.ensembl.org/index.html). Source data are provided with this paper.

Research involving human participants, their data, or biological material

Policy information about studies with human participants or human data. See also policy information about sex, gender (identity/presentation), and sexual orientation and race, ethnicity and racism.

Reporting on sex and gender	n/a
Reporting on race, ethnicity, or other socially relevant groupings	n/a
Population characteristics	n/a
Recruitment	n/a
Ethics oversight	n/a

Note that full information on the approval of the study protocol must also be provided in the manuscript.

Field-specific reporting

Please select the one below that is the best fit for your research. If you are not sure, read the appropriate sections before making your selection.

☒ Life sciences ☐ Behavioural & social sciences ☐ Ecological, evolutionary & environmental sciences

For a reference copy of the document with all sections, see nature.com/documents/nr-reporting-summary-flat.pdf

Life sciences study design

All studies must disclose on these points even when the disclosure is negative.

Sample size

We follow related studies and instructions for standard protocols to determine the sample size. For qRT–PCR , at least three independent samples were analyzed. For HI testing, at least 300 plants derived from each cross/selfing were screened. The sample size is sufficient as determined in related studies. We have cited the references in the manuscript.

18. Sanei, M et al. Loss of centromeric histone H3 (CENH3) from centromeres precedes uniparental chromosome elimination in interspecific barley hybrids. Proc. Natl. Acad. Sci. USA 108, E498–E505 (2011).
19. Karimi-Ashtiyani, R. et al. Point mutation impairs centromeric CENH3 loading and induces haploid plants. Proc. Natl. Acad. Sci. USA 112, 11211–11216 (2015).
21. Lv, J. et al. Generation of paternal haploids in wheat by genome editing of the centromeric histone CENH3. Nat. Biotechnol. 38, 1397–1401 (2020).
22. Wang, N. et al. Haploid induction by a maize cenh3 null mutant. Sci. Adv. 7, eabe2299 (2021).

Data exclusions

No data was excluded from the analyses.

Replication

We conducted at least three biological replicates for each experiment. These information has been described in the methods or relevant figure legends.

Randomization

Plants used in this study were randomly grown in the same open field, greenhouse or growth chamber. Plants samples were collected randomly.

Blinding

Researchers were blinded during ploidy level detection. Researchers were blinded during plant cultivation, qRT–PCR, phylogenetic analyses, CRISPR/Cas9-mediated gene editing, genome resequencing, genotyping and HI rate calculation as blinding is not a necessary experimental design in plant sciences.

Reporting for specific materials, systems and methods

We require information from authors about some types of materials, experimental systems and methods used in many studies. Here, indicate whether each material, system or method listed is relevant to your study. If you are not sure if a list item applies to your research, read the appropriate section before selecting a response.

Materials & experimental systems

n/a	Involved in the study
☒	☐ Antibodies
☒	☐ Eukaryotic cell lines
☒	☐ Palaeontology and archaeology
☒	☐ Animals and other organisms
☒	☐ Clinical data
☒	☐ Dual use research of concern
☐	☒ Plants

Methods

n/a	Involved in the study
☒	☐ ChIP-seq
☐	☒ Flow cytometry
☒	☐ MRI-based neuroimaging

Dual use research of concern

Policy information about dual use research of concern

Hazards

Could the accidental, deliberate or reckless misuse of agents or technologies generated in the work, or the application of information presented in the manuscript, pose a threat to:

No	Yes
☒	☐ Public health
☒	☐ National security
☒	☐ Crops and/or livestock
☒	☐ Ecosystems
☒	☐ Any other significant area

Experiments of concern

Does the work involve any of these experiments of concern:

No	Yes
☒	☐ Demonstrate how to render a vaccine ineffective
☒	☐ Confer resistance to therapeutically useful antibiotics or antiviral agents
☒	☐ Enhance the virulence of a pathogen or render a nonpathogen virulent
☒	☐ Increase transmissibility of a pathogen
☒	☐ Alter the host range of a pathogen
☒	☐ Enable evasion of diagnostic/detection modalities
☒	☐ Enable the weaponization of a biological agent or toxin
☒	☐ Any other potentially harmful combination of experiments and agents

Plants

Seed stocks: n/a

Novel plant genotypes: We used CRISPR/Cas9 system to generate BoCENH3 mutants. Information of targets for editing, sgRNAs and application of the editor was provided within this paper.

Authentication: We used broccoli inbred line CX33 to generate BoCENH3 mutants. We assessed the phenotypes, seed setting, and cross pollinations of the BoCENH3 mutants and their wild type plants.

Flow Cytometry

Plots

Confirm that:

- [x] The axis labels state the marker and fluorochrome used (e.g. CD4-FITC).
- [x] The axis scales are clearly visible. Include numbers along axes only for bottom left plot of group (a 'group' is an analysis of identical markers).
- [ ] All plots are contour plots with outliers or pseudocolor plots.
- [x] A numerical value for number of cells or percentage (with statistics) is provided.

Methodology

Sample preparation: Young leaves were sampled, and subjected to lysate treatment to release nuclears. The samples were subsquently centrifuged with 1000rpm and dyed for 30 min.

Instrument: BD LSRFortessaTM Flow Cytometer, BD Biosciences, San Jose, CA, USA

Software: LSRFortessa

Cell population abundance: By centrifuge

Gating strategy: Bulk cell population

- [ ] Tick this box to confirm that a figure exemplifying the gating strategy is provided in the Supplementary Information.
