# Supplementary figures and images for: One-step creation of CMS lines using a BoCENH3-based haploid induction system in Brassica crop
Source: Nat Plants. 2024 Mar 18;10(4):581–6. doi: 10.1038/s41477-024-01643-w (PMC11035129; doi:10.1038/s41477-024-01643-w)

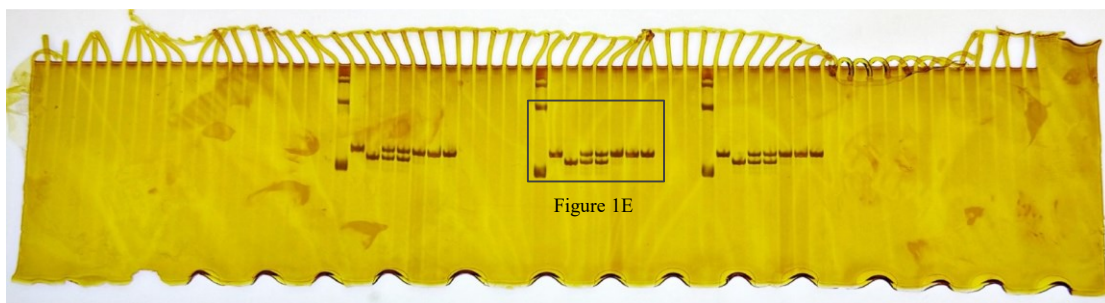

Figure 1E

Supplement: Supplementary file 6 — Unprocessed gels for Fig. 1e. [file 41477_2024_1643_MOESM6_ESM.pdf]

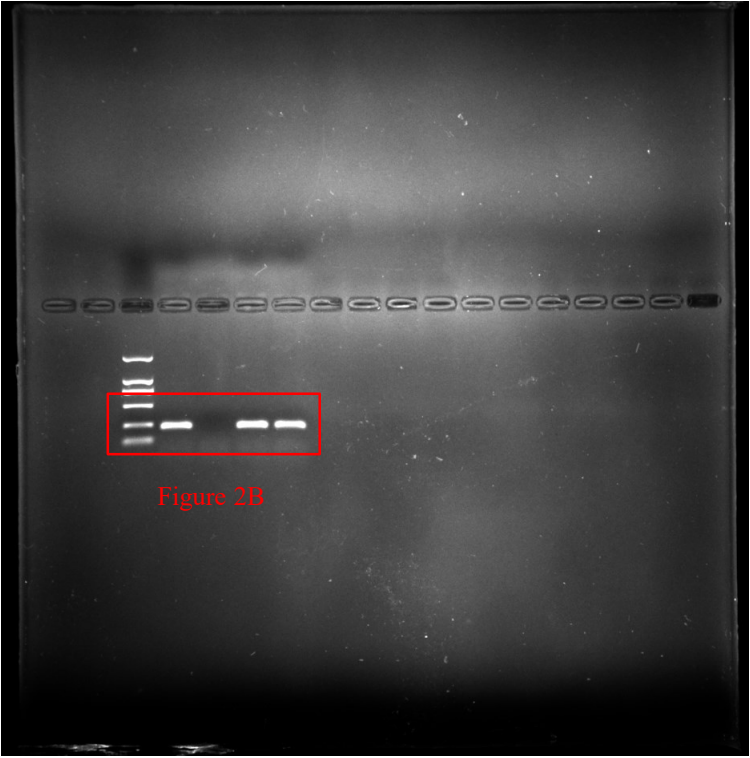

Figure 2B

Supplement: Supplementary file 7 — Unprocessed gels for Fig. 2b. [file 41477_2024_1643_MOESM7_ESM.pdf]
